# Comparıson of analgesıc effectıveness of ultrasound-guıded caudal epıdural block and transversus abdomınıs plane block ın chıldren undergoıng unılateral ınguınal hernıa repaır: randomized controlled study

**DOI:** 10.1007/s10029-026-03633-7

**Published:** 2026-03-16

**Authors:** Mustafa Kaçmaz, Özlem Yandım

**Affiliations:** 1 Department of Anesthesiology, Training and Research Hospital, Nigde, Turkey; 2 Department of Pediatric Surgery, Faculty of Medicine, Ömer Halisdemir University, Nigde, Turkey

**Keywords:** Caudal block, Transversus abdominis plane block, Inguinal hernia surgery, Pediatric analgesia

## Abstract

**Objectives:**

The introduction of ultrasound-guided nerve blocks has led to significant advancements in pediatric postoperative analgesia. In this context, the caudal block (C), a well-established and effective analgesic technique used for many years, has been increasingly complemented by the transversus abdominis plane (TAP) block, which is thought to have fewer side effects and potentially greater efficacy. The aim of our study was to compare these two methods in terms of postoperative analgesic effectiveness.

**Materials and methods:**

This study was conducted as a prospective, randomized, controlled trial. A total of 56 children, aged 1 to 10 years, classified as ASA I and II, undergoing elective inguinal hernia repair, were included. The patients were randomly assigned into two groups. Group T (n = 28) received a TAP block with 0.5 mL/kg of 0.25% bupivacaine under ultrasound guidance, while Group C (n = 28) received a caudal block with the same concentration and volüme of bupivacaine.

The primary outcome of the study was the duration of postoperative analgesia, while secondary outcomes included the need for rescue analgesics, complications, and the effects on hemodynamic parameters.

**Results:**

During the postoperative period, adequate analgesia was achieved in both groups for up to the first 4 h. However, after the 6th hour, there was a significant increase in pain scores in the caudal block group. The need for rescue analgesics was lower in the TAP group, although there was no difference in the total amount of analgesics used at 12 h.

**Conclusion:**

Both TAP block and caudal block are effective in providing postoperative analgesia for children undergoing inguinal hernia repair. The TAP block may be preferred due to its longer-lasting postoperative effect and lower need for rescue analgesics.

## Introductıon

Inguinal hernias are a common health issue in the population. Diagnosis is typically made through physical examination and patient history, with imaging rarely required. In cases of minimal symptoms, a period of watchful waiting and observation may be preferred. However, there is also a risk of incarceration and strangulation of the hernia. Therefore, in elective conditions, surgical repair is generally needed as soon as possible for the operation. Inguinal hernia repair is usually a safe surgical procedure; nevertheless, postoperative pain can sometimes pose a significant problem [1].

In the postoperative management of analgesia following inguinal hernia surgery, pharmacological treatment plays a crucial role during the early recovery period. To this end, conventional non-steroidal anti-inflammatory drugs (NSAIDs) or selective cyclooxygenase-2 (COX-2) inhibitors, along with paracetamol, are commonly used in combination. Additionally, weak opioids are employed for moderate pain, while strong opioids continue to be used for severe pain [2].

In the surgical repair of inguinal hernia in children, postoperative pain management is significantly enhanced by regional anesthesia. To this end, various techniques have been employed, including the caudal block, transversus abdominis plane block, quadratus lumborum block, paravertebral block (PVB), retrolaminar block, and ilioinguinal/iliohypogastric nerve blocks. While the paravertebral block has also emerged as an effective method for alleviating acute postoperative pain, its technical complexity and potential for serious complications may cause hesitation among practitioners. The transversus abdominis plane block (TAPB), on the other hand, may be preferred due to its potential for longer-lasting analgesic effects [3]

The caudal epidural block (CEB) is a well-known, safe, and effective neuroaxial analgesic technique for lower abdominal surgeries. However, alongside unintended dural puncture, perhaps its least favorable side effect is the dose-dependent occurrence of prolonged motor block and urinary retention due to impaired bladder function. When performed as a single-shot technique, another limitation of a complication-free CEB is its short duration of effect (up to 6 h), necessitating the administration of additional analgesics [4, 5].

With the increasing adoption of ultrasound practice, practitioners’ experience in this field has also advanced. Through ultrasound guidance, the real-time visualization of muscle layers and fascial planes significantly simplifies the application of regional nerve blocks, as ultrasound confirms the correct spread of the local anesthetic (LA) in the intended area [6]. Ultrasound-guided TAP block, in particular, allows for the visual confirmation of the blockade of spinal afferent nerves (T7-L1) within the neurofascial plane between the internal oblique and transversus abdominis muscles. This not only enhances the effectiveness of the block but can also lead to a significant reduction in pain intensity and analgesic requirements [7].

Therefore, since it has emerged as a valid postoperative analgesia alternative in adults undergoing abdominal surgery, interest in ultrasound-guided transversus abdominis plane (TAP) block has been rekindled in pediatric patients. In the postoperative pain management of pediatric inguinal hernia repair, both TAPB and CEB stand out due to their dose-dependent effects and side effects. The drug doses commonly used for pediatric TAPB are generally 0.5 mL/kg, whereas for caudal block, doses typically range from 0.5 to 1 mL/kg [8].

The aim of our study is to compare, in terms of postoperative analgesic effectiveness and complications, the TAP block performed with standard volumes under ultrasound guidance and the caudal block similarly applied with ultrasound guidance.

## Materıals and methods

This study was conducted at a tertiary healthcare center with the approval of the Institutional Ethics Committee (Reference No. 2022/99, dated 27.10.2022) and with the written informed consent of the parents of the participating children.

**Fig. 1:**
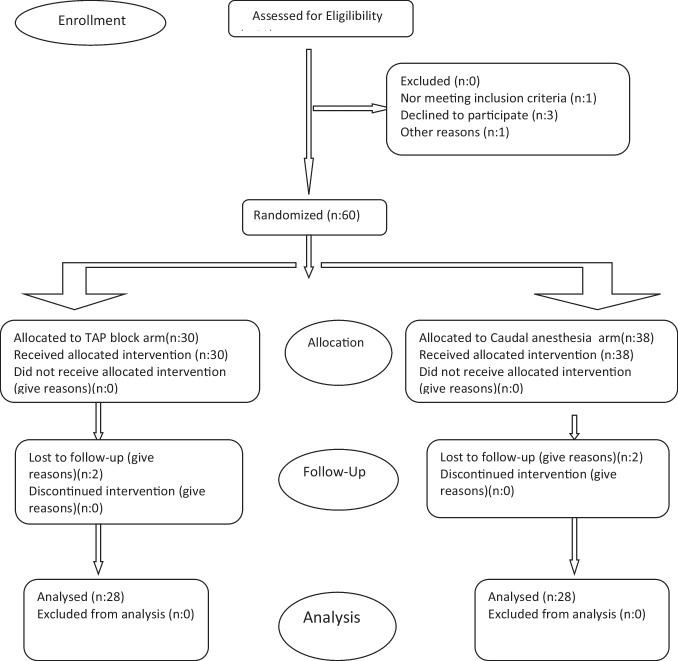
CONSORT 2010 flow diagram

A total of 56 patients were included in the study, all of whom were scheduled to undergo unilateral elective inguinal hernia repair at our hospital’s pediatric surgery clinic between February 2023 and May 2025. The patients had an ASA physical status of I-II and were aged between 1 and 10 years. The patients were randomized into two groups, each consisting of 28 patients, using a computer-generated random number table (Fig. 1).

In the Group T patients: While the children were in the supine position, a TAP block was administered under ultrasound guidance using 0.5 mL/kg of 0.25% bupivacaine.

In the Group C patients: While the children were positioned on their left side, a caudal block was planned under ultrasound guidance using 0.5 mL/kg of 0.25% bupivacaine.

The exclusion criteria were as follows: children who underwent bilateral inguinal hernia surgery, children who underwent additional surgical procedures in anatomical regions not covered by the TAP block or CEB, children with a known allergy to bupivacaine, children with a history of kidney and/or liver failure, children with pre-existing cardiac dysfunction, lack of parental consent, coagulation disorders, local infections, and spinal deformities.

All patients underwent a comprehensive preoperative examination at least one day before the surgery. Upon arrival in the operating room, the patient received premedication with 0.5 mg/kg of oral midazolam 30 min prior to the surgery in the preoperative medication room.

Subsequently, the patient was taken into the operating room, and monitoring of EKG, pulse oximetry, and non-invasive arterial blood pressure was initiated. An intravenous line was established, and a 1/3 isotonic solution (each 100 mL of the solution containing dextrose monohydrate and sodium chloride) was administered. General anesthesia induction was achieved following preoxygenation with 100% O2, and within 20–30 s, 1.5–2.5 mg/kg of propofol, 0.5 mg/kg of rocuronium to facilitate endotracheal intubation, and 2 μg/kg of fentanyl were administered. The patients were intubated with an appropriately sized endotracheal tube. Anesthesia was maintained with an O2-to-air ratio of 40:60 and sevoflurane (1–2%), and, if necessary, 0.15 mg/kg of rocuronium was administered.

**Fig. 2:**
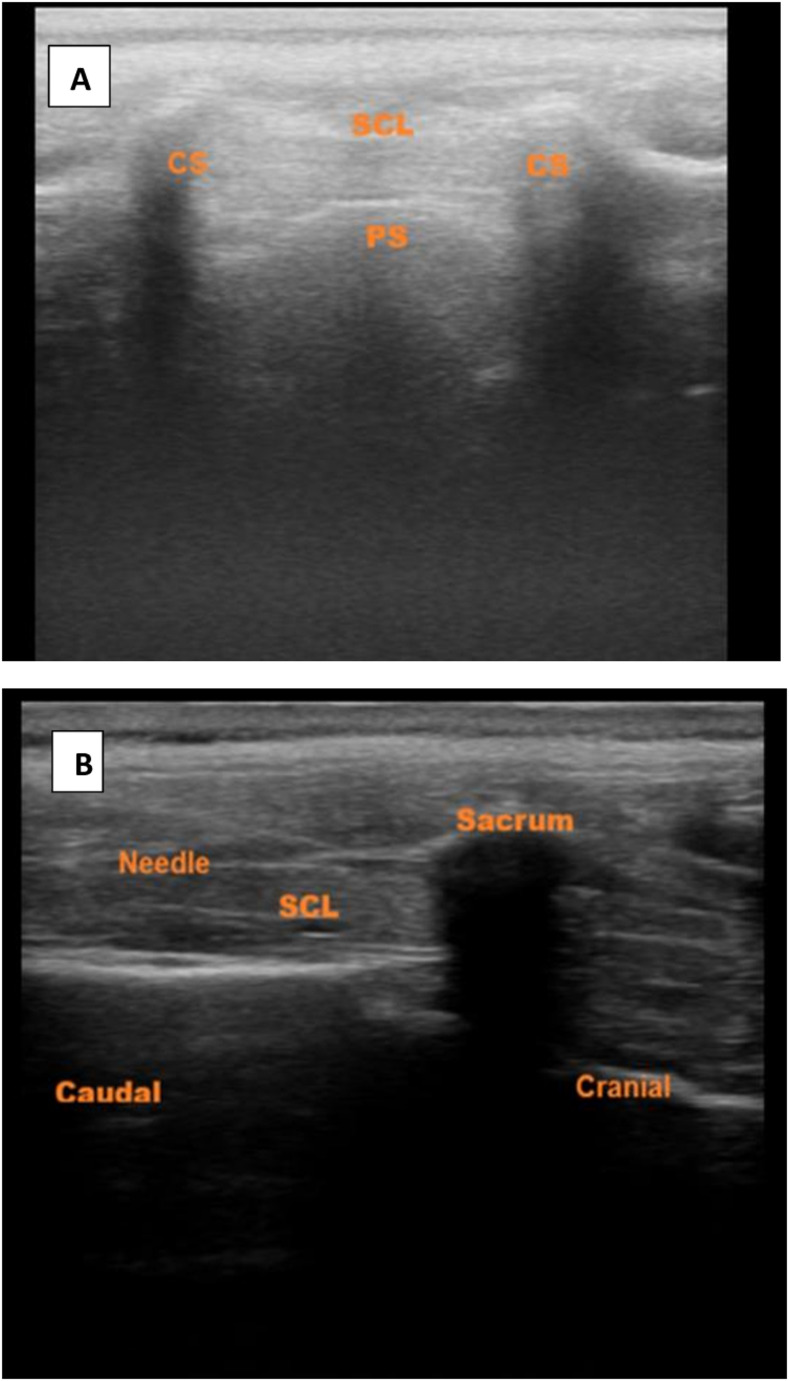
**A** Caudal İnjection (out of plane image), **B**: Caudal Injection (in plane image). BN: block needle; CS: cornu of sacrum; PS: posterior surface of sacrum; S: sacrum; SC: sacral canal; SL: sacrococcygeal ligament

Immediately after induction, in Group C, after taking all aseptic precautions and achieving sterilization of the area in the left lateral decubitus position, the sacral cornua and hiatus were visualized using a portable ultrasound device (Samsung HM70 EVO) with a linear US probe.The probe was then rotated 90 degrees to visualize the sacrococcygeal ligament and the caudal canal (Fig. 2). Using the in-plane technique, a 20–22 gauge needle was inserted through the skin above the sacrococcygeal ligament. The needle tip was continuously visualized in real time until it entered the sacral canal. After confirming the absence of blood or cerebrospinal fluid upon aspiration, 0.5 ml/kg of 0.25% bupivacaine was injected (Fig. 2).

**Fig. 3:**
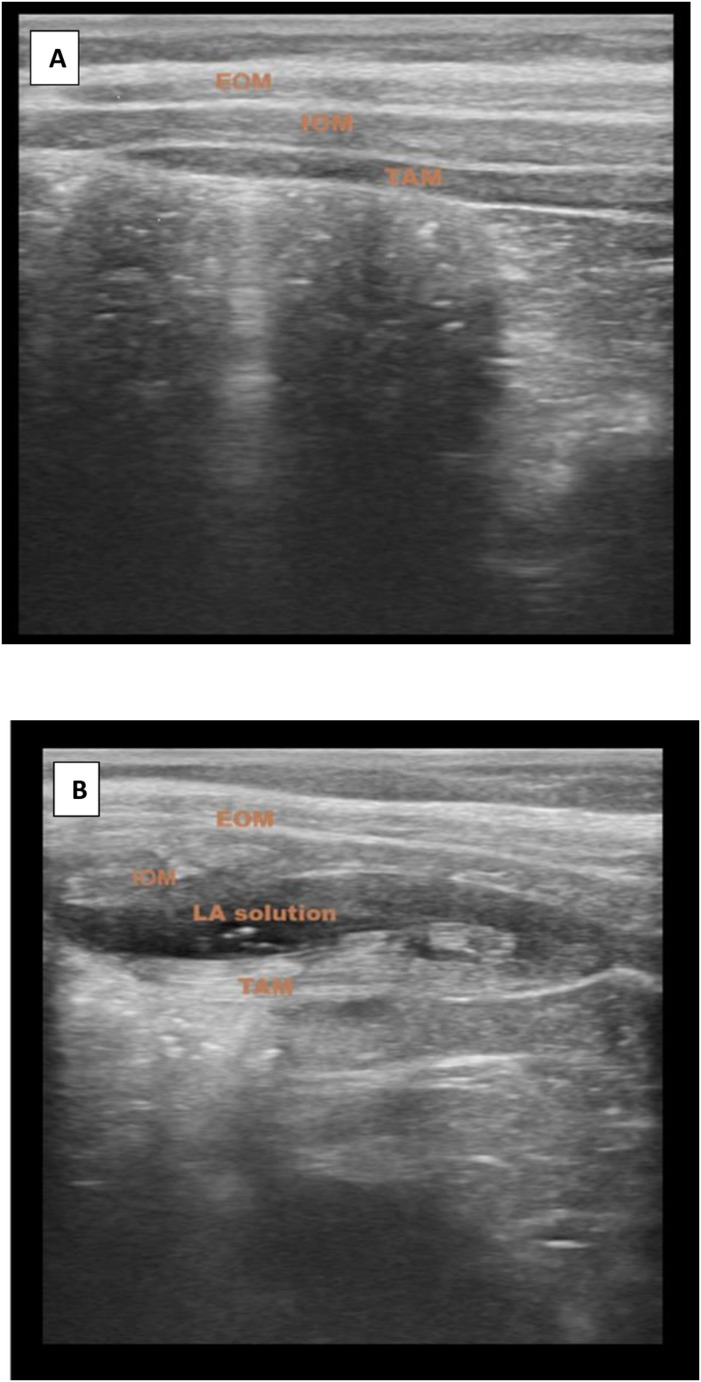
**A** Tap Block USG image (before injection). **B** Tap Block USG image (distribution of local anesthetic after injection). Ultrasonography image showing the muscle layers of the abdominal wall. EOM: External oblique muscle, IOM: Internal oblique muscle, TAM: Transversus abdominis muscle, LA: Local anesthetics

In Group T, the patients were positioned supine with all aseptic precautions taken. The needle entry site was sterilized. The abdominal wall was then scanned using a portable ultrasound device (Samsung HM70 EVO) with a linear transducer probe (6–13 MHz). Initially, the rectus abdominis muscle was visualized, and then the scan was moved laterally. The internal oblique and transversus abdominis muscles were then visualized, and a 22-G short-beveled 50 mm block needle was advanced, in the same plane as the transducer, between the fascial sheath of the internal oblique and transversus abdominis. Subsequently, 0.5 mL/kg of 0.25% bupivacaine was administered. The correct spread of the local anesthetic was demonstrated by a hypoechoic elliptical fluid image between these two muscles (Fig. 3).

In both groups, the procedure durations were recorded. The surgery was initiated 10 min after the administration of the caudal block or the TAP block. During the intraoperative period, blood pressure, pulse, SpO2 values, and the duration of the surgery were documented. Postoperatively, the patient’s initial pain assessment was conducted at the second hour. The block was performed by an anesthesiologist experienced in this procedure, and this anesthesiologist did not participate in the pain assessment. All surgeries were performed by a single pediatric surgeon with extensive experience in the field.

The initial need for rescue analgesia, the time of mobilization, the total analgesic dose, and complications (such as hypotension, bradycardia, respiratory depression, urinary retention, and postoperative nausea and vomiting) were observed. Patients with CHEOPS scores ≥ 6 received 10 mg/kg of intravenous paracetamol syrup as rescue analgesia.

**Fig. 4:**
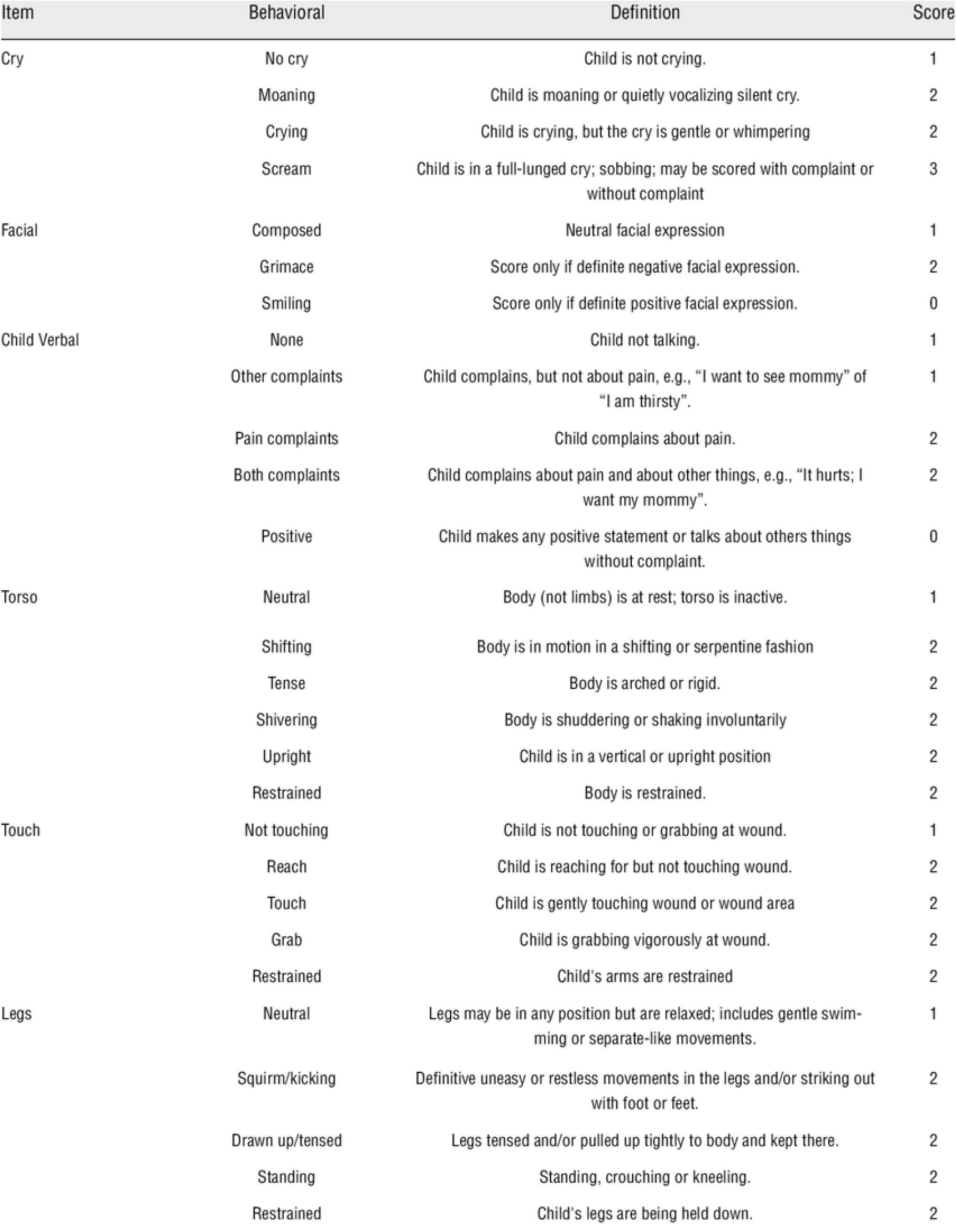
CHEOPS Score

In our study, we used the Children’s Hospital Eastern Ontario Pain Scale (CHEOPS) to measure postoperative pain. CHEOPS was developed in 1985 by McGrath and colleagues [9]. When adapted to our country, it was found to have high levels of validity and reliability. [10] The scale includes six categories of pain behaviors: crying, facial expression, verbal cues, torso, touch, and legs. Each category has three or four levels. The minimum possible score for CHEOPS is 4 points (no pain), and the maximum score is 13 points (most severe pain) (Fig. 4).

### Statıstıcs

Statistical analysis was performed using SPSS for Windows, version 21.0 (SPSS, Chicago, IL). Continuous variables are presented as mean ± standard deviation or median and interquartile range, while categorical variables are presented as absolute numbers and percentages.

Before statistical analysis, the normality of the data was checked. For continuous variables with normal distribution, comparisons were made using the unpaired t-test, while the Mann–Whitney U test was used for non-normally distributed variables. Categorical variables were analyzed using the χ2 test or Fisher’s exact test. A p-value of < 0.05 was considered statistically significant for all tests.

The sample size calculation was performed using G*Power (version 3.1.9.4) for a noninferiority study design. A 95% confidence level and a 9.5% confidence interval were assumed. The clinically significant difference was set at a 10% noninferiority margin, based on the literature. Under these assumptions, it was calculated that 23 patients per group would be sufficient to demonstrate noninferiority in the comparison of ultrasound-guided transversus abdominis plane (TAP) block and caudal analgesia.

## Results

This study included fifty-six patients who underwent elective unilateral open inguinal hernia surgery. In Group T, in 28 patients, the abdominal muscles, needle placement, and the spread of the local anesthetic were clearly observed, and the TAP block was successfully performed. In Group C, in 28 patients, the sacral cornua and hiatus were visualized, and the caudal block was successfully performed. There was no significant difference between the two groups in terms of demographic characteristics (age, weight, gender, surgical side, and ASA score) (Table 1).

**Table 1:** Age, weight, height, ASA and gender characteristics of groups

	**GROUP (1)** **(Caudal)**	**GROUP (2)** **(TAP)**	
**Age**^**b**^ **(months)**	24.57 (24–105)	32.43(22–95)	0.071
**Height**^**a**^ (cm)	105.07 ± 12.78	112.25 ± 11.97	0.067
**Weight **^**b**^ **(kg)**	25,32(10–32)	31,68 (14–53)	0.143
**ASA**^**c**^ **(I/II)**	26/2	27/1	0.556
**Gender **^**c**^ **(M/F)**	19/9	12/16	0.125
**Surgical Side **^**c**^ **Right/left**	20/8	20/8	0.587

Data presented as mean SD or number of patients (%). ^a^ Student-T test.^b^Mann–Whitney U-test. ^c^Pearson’s 2 –test.^d^ Fisher’s exact test

Statistically significant between-group differences (P < 0.05) *

ASA, American Society of Anesthesiologists

f = female, m = male

There was no significant difference between the groups in terms of surgical duration, discharge time, initial mobilization time, procedure duration, and total analgesic dose used. However, the time to first analgesic was statistically significantly earlier in Group C. In Group C, 28 patients, and in Group T, 21 patients, required rescue analgesia at some point during the twelve-hour follow-up, and this difference was statistically significant. There was no statistically significant difference in the total amount of analgesics used. Complications such as nausea or vomiting occurred in one patient in Group C and two patients in Group T, but this was not statistically significant. Moreover, no motor block or urinary retention occurred in any patient in Group C (Table 2).

**Table 2:** Duration of surgery,analgesic

	**GROUP (1)** **(Caudal)**	**GROUP (2)** **(TAP)**	
**Duration of** ^a^ **surgery (Min)**	31.32 ± 7.97	34.61 ± 8.17	0.205
**Duration of** ^b^ **First Analgesic**	3.00 (0–12)	6.00(0–12)	0.001
**Duration of Discharge**^b^	4.51(3–7)	5.07 (3–9)	0.124
**Duration of** ^b^ **First Mobilization**	3.75(2–6)	4.14 (2.5–6)	0.225
**Toal Analgesic Dose **^**b**^	181.43 (0–260)	182.50 (0–450)	0.811
**Procedure Time**^**b**^	9 (3–13)	9.54 (5–15)	0.381
**Complication**^**d**^ **No/Yes**	27/1	26/2	1.000

Data presented as mean SD or number of patients (%). ^a^ Student-T test.^b^Mann–Whitney U-test. ^c^Pearson’s 2 –test.^d^ Fisher’s exact test

Statistically significant between-group differences (P < 0.05) *

ASA, American Society of Anesthesiologists

f = female, m = male

**Fig. 5:**
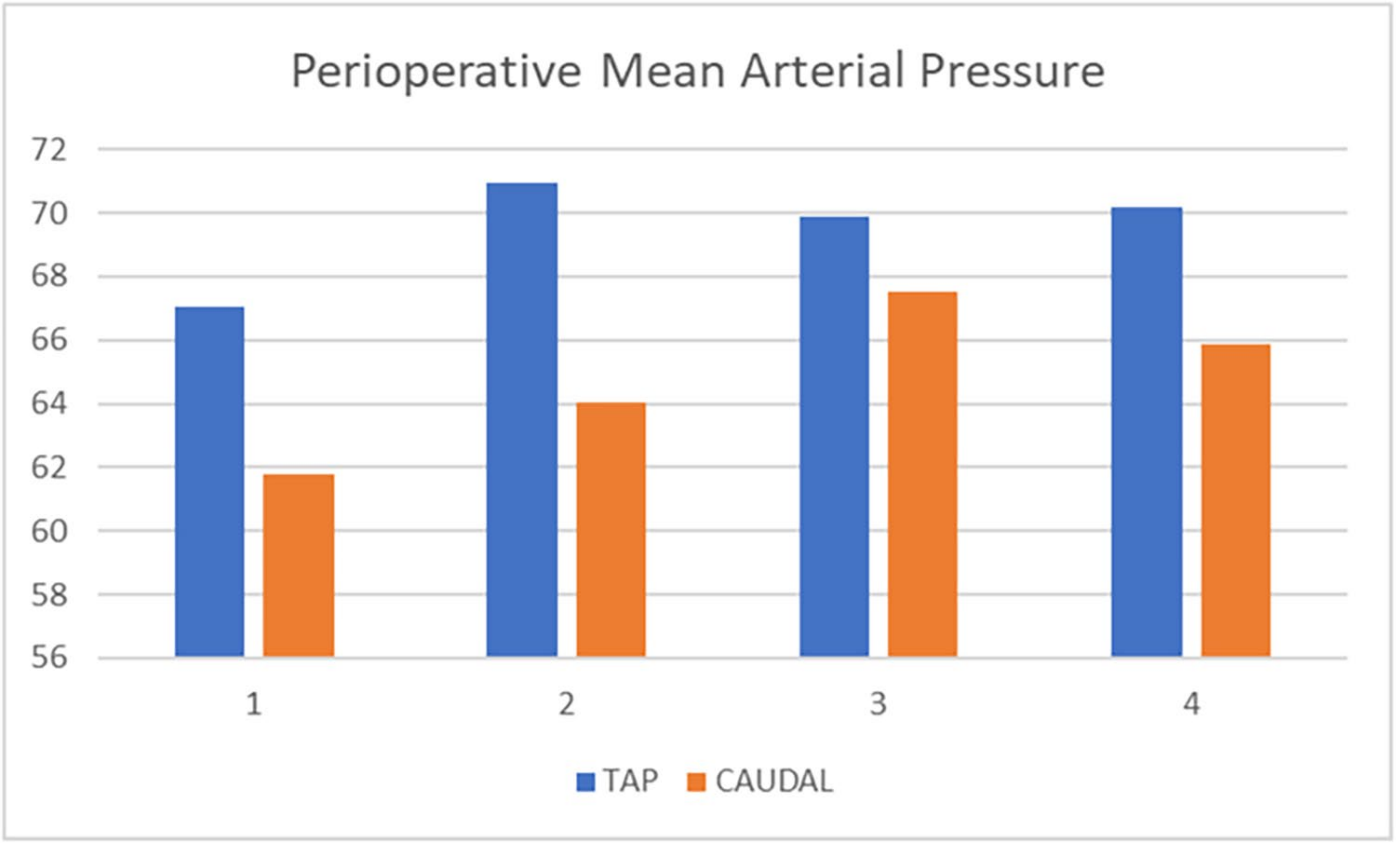
Change in Mean Arterial Pressure Over Time. 1: at skin incision; 2: 15 min thereafter (after skin incision); 3: 30 min thereafter; 4: 45 min thereafter

Intraoperative hemodynamic parameters (heart rate, systolic blood pressure, diastolic blood pressure, and mean arterial pressure) remained within normal limits and showed no significant change (> %20) compared to baseline in both groups. Furthermore, no significant difference was found between the groups in terms of SpO2 values at the start of the surgery, and at 15, 30, and 45 min. Regarding OAB values, these were significantly higher in the TAP group at baseline and at 15 min, but there was no difference at 30 and 45 min. (Fig. 5) Heart rate values, on the other hand, were consistently higher in Group C at all measurement times (Table 3).

**Table 3:** Comparison of SPO2, MAP, PULSE values

	**GROUP (1)** **(Caudal)**	**GROUP (2)** **(TAP)**	
**SPO2** ^b^ **(%)**			
**0 min**	98.43(83–100)	98.68 (93–100)	0.659
**15 min**	99.14(98–100)	98.79(95–100)	0.291
**30 min**	99.04(96–100)	99.00(97–100)	0.663
**45 min**	99.09(98–100)	99.00(98–100)	0.831
**MAP **^**a**^ **(mm/hg)**			
**0 min**	61.78 ± 7.79	67.02 ± 9.31	0.026*
**15 min**	64.01 ± 9.51	70.94 ± 8.85	0.007*
**30 min**	67.50 ± 9.01	69.88 ± 9.60	0.344
**45 min**	65.83 ± 9.82	70.20 ± 7.73	0.070
**PULSE**^**a**^			
**0 min**	108.46 ± 16.65	98.93 ± 13.59	0.023*
**15 min**	103.43 ± 20.79	91.93 ± 15.61	0.023*
**30 min**	103.25 ± 18.39	89.86 ± 14.93	0.004*
**45 min**	100.61 ± 15.10	89.07 ± 11.96	0.004*

Data presented as mean SD or number of patients (%). ^a^ Student-T test.^b^Mann–Whitney U-test. ^c^Pearson’s 2 –test.^d^ Fisher’s exact test

Statistically significant between-group differences (P < 0.05) *

ASA, American Society of Anesthesiologists f = female, m = male

**Fig. 6:**
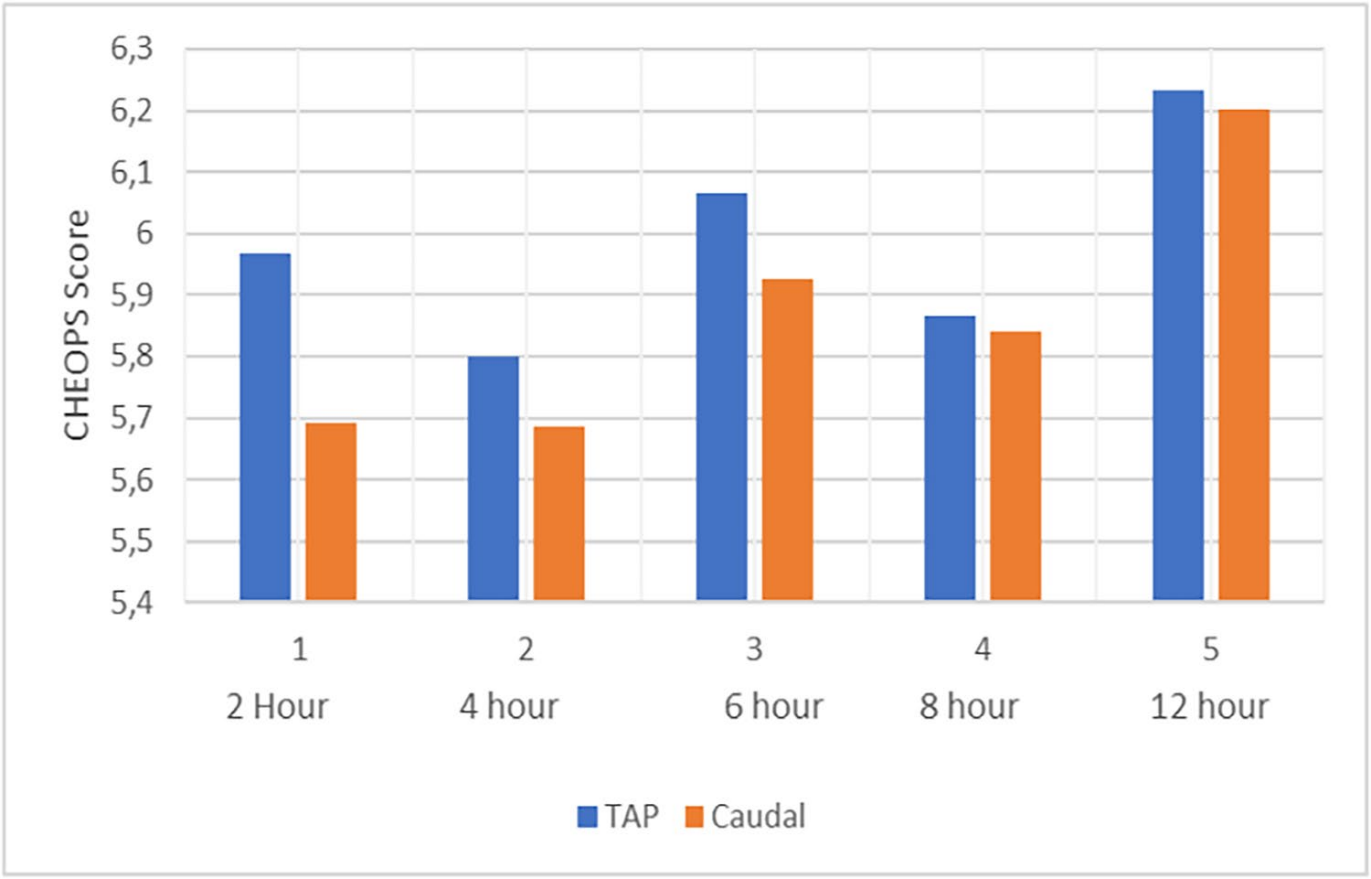
CHEOPS scores by time

In the assessments of CHEOPS scores at the 2nd, 4th, 6th, 8th, and 12th hours, no significant difference was observed between the groups at the 2nd and 4th hours (Fig. 6). However, at the 6th, 8th, and 12th hours, the scores in the caudal anesthesia group were found to be significantly higher (Table 4).

**Table 4:** Comparison of CHEOPS pain scores

	**GROUP (1)** **(Caudal)**	**GROUP (2)** **(TAP)**	
**Pain Score**^a^			
2 h	5.50 (4–11)	6.00 (4–11)	0.293
4 h	5.71 (4–9)	5.86(4–11)	0.539
6 h	7.14(6–10)	6.04 (4–10)	0.000
8 h	6.71 (5–9)	5.89 (4–9)	0.002
12 h	7.96 (6–9)	6.25 (6–9)	0.000

Data presented as mean SD or number of patients (%). a Student-T test b Mann–Whitney U-test. c Pearson’s 2 -test. d Fisher’s exact test.) * CHEOPS Score.h: hour

Statistically significant between-group differences (P < 0.05) * h: hours

## Dıscussıon

Perioperative anesthesia techniques are frequently used in infants and children for abdominal and lower extremity surgeries. Among these regional anesthesia techniques, the TAP block has been shown to reduce opioid consumption and improve pain scores compared to traditional perioperative pain management strategies. Therefore, TAP blocks are becoming increasingly popular as an alternative to pediatric neuroaxial analgesia methods for optimizing perioperative pain control. Despite studies indicating the potential benefits of TAP blocks, their use is not yet widespread, particularly among pediatric anesthesiologists [11].

Caudal anesthesia continues to be widely used for providing analgesia after surgeries involving the lower abdomen, pelvis, genital region, or lower extremities. When using CEB, it is important to anticipate a mild or moderate systemic analgesic requirement to prevent the recurrence of pain once the block effect wears off.

During caudal anesthesia, complications such as dural puncture, vascular nerve injury, cardiovascular issues due to overdose, unwanted motor block, and urinary retention may occur. In particular, motor block and urinary retention associated with higher doses tend to be more frequent and contribute to a negative patient experience [12].

Kumar A et al. compared these two techniques in terms of postoperative analgesic efficacy, complications, and the need for rescue analgesia, and reported no difference in surgical duration. They observed a higher requirement for rescue analgesics in the caudal group and noted that pain scores in the caudal group increased from the 6th postoperative hour onward. In our study, no differences were observed between the two techniques with respect to surgical duration, procedure time, discharge time, time to first mobilization, total analgesic dose, or complications. However, the time to first analgesic administration was earlier in the caudal group. Rescue analgesia was required in 21 patients in the TAPB group, whereas all patients in the CEB group required rescue analgesics during follow-up [13].

Wafaa Mohamed Alsadek et al. compared TAP block and caudal anesthesia in their study. They observed a significant increase in postoperative CHEOPS scores in the caudal group starting from the 6th postoperative hour, whereas the analgesic effect in the TAP group was maintained up to the 12th hour. In our study, a difference in analgesic efficacy between the TAP and caudal groups also emerged from the 6th postoperative hour onward. Our findings are consistent with these results [14].

Ashraf A. et al. evaluated two groups of 20 patients each who received TAP block and caudal analgesia and reported no differences in surgical duration, intraoperative mean arterial pressure (MAP), SpO₂, or heart rate values, nor in postoperative analgesic efficacy during the first four hours of follow-up (15). In our study, while no difference was observed in SpO₂ values between the groups, heart rate was higher in the CEB group, and mean arterial pressure was higher in the TAP group.

We believe that the higher MAP observed in the TAPB group may be related to the slightly higher mean age (measured in months) in this group, although this difference was not statistically significant. The higher intraoperative heart rate values in the C group may be attributable to the earlier onset of analgesic efficacy in the TAP group. In our study, a difference in postoperative analgesic efficacy between the two groups became evident from the 6th postoperative hour onward. This difference may not have been observed by Ashraf et al. because their postoperative follow-up was limited to four hours [15].

Nitin Sethi et al. compared TAP block and caudal block in lower extremity surgery and reported that, despite a higher requirement for rescue analgesia in the caudal group, there was no difference in total analgesic consumption or pain scores between the groups. Similarly, in our study, the need for rescue analgesia was greater in the CEB group, while no difference was observed in total analgesic dose. However, in the postoperative period, pain scores were higher in the CEB group from the sixth hour onward [16].

V. Rajesh Kumar Kodali et al. reported no difference in surgical duration after administering a TAP block with 0.5 mL/kg and a caudal block with 1 mL/kg bupivacaine. They observed that the time to first analgesic requirement occurred earlier in the CEB group and that total analgesic consumption was higher in this group. Pain scores in the CEB group, which began to increase from the sixth postoperative hour, reached statistical significance at the 12th and 18th hours. These findings are consistent with the results of our study [17].

Hayder Abbas et al., in their study comparing TAPB and CEB in inguinal hernia surgery, reported a higher requirement for rescue analgesics in the caudal group; however, postoperative pain intensity was lower in the CEB group up to the seventh hour. In our study, no difference was observed between the groups during the first six hours, whereas after the sixth hour, pain scores were lower in the TAP group. Consistent with this, we did not find a difference in pain scores between the groups during the first six postoperative hours [18].

Different concentrations and volumes of local anesthetics can be used for caudal blockade. Silvani P. et al. reported that, in children undergoing hypospadias repair, a caudal block administered using a “high-volume, low-concentration” regimen provided longer-lasting analgesia and less motor blockade compared with a “low-volume, high-concentration” regimen. In our study, we administered bupivacaine at a dose of 0.25% and 0.5 mL/kg for the caudal block. This volume is lower than the commonly used 1 mL/kg, while the concentration represents a moderate level [19].

## Conclusıon

In our study, we observed that both TAP block and caudal epidural block (CEB) anesthesia techniques applied in the early postoperative period were effective in providing postoperative analgesia in patients undergoing unilateral inguinal hernia surgery.

Neither technique demonstrated superiority over the other in the early postoperative period. A caudal block administered with 0.25% bupivacaine at a dose of 0.5 mL/kg was sufficient to provide early postoperative analgesia without causing motor block or urinary retention. However, CEB may be inadequate in maintaining analgesic efficacy beyond six hours, whereas the TAP block may provide longer-lasting postoperative analgesia and reduce the need for rescue analgesics.

Author Contributions All authors meet ICMJE criteria: MK, ÖY- study concept, design, data analysis, manuscript draft; MK -, analysis; MK, ÖY; MK, ÖY—data collection, critical revision; MK—senior supervision,critical revision, final approval. All authors have approved the final manuscript and agree to be responsible for all aspects of the study.

Funding Open access funding provided by Ömer Halisdemir University. This research has not received any private grants from funding organizations in the public, commercial, or non-profit sectors.

## Declarations

### Artificial Intelligence

No artificial intelligence tools were used in the study design, data collection, analysis, or manuscript drafting. All writings and analyses represent the original work of the authors.
